# Phrase of attentiveness: Use of smart phone applications for mental health concerns

**DOI:** 10.1016/j.amsu.2022.104783

**Published:** 2022-09-25

**Authors:** Hareem Arshad, Samiullah Dahri, Muhammad Yusuf Hafiz

**Affiliations:** Jinnah Sindh Medical University (JSMU), H J Rafiqui Road, Shahrah e Faisal, Karachi, Pakistan; Shaheed Mohtarma Benazir Bhutto Medical College, Near Lyari General Hospital, Tannery Road, Lyari, Karachi, Pakistan; Dow University of Health Sciences, Baba e Urdu Road, Karachi, Pakistan

Mam/Sir,

In the aftermath of the COVID-19 pandemic, there is an immense increment in the use of smartphone applications to assess, diagnose and treat mental health issues. Isolation and segregation due to the global pandemic that led to confinement inside homes, severe mental health concerns came into attention, and there is an enormous supplementation in the conversations around the topic globally, with the lack of provision of facilities more pronounced in an underdeveloped world in comparison to developing countries. Stigmatized mental illnesses have started to be given due importance, which has increased the demand for mental health therapists who can deal with uprising ailments. This supply demand mismatch has given birth to digital psychiatry in the form of digital smartphone applications that can be used by anyone across the globe and can connect people regardless of borders, race; color; religion; or creed. It's a provision of mental health at the doorstep without caring much about mobility for the assessment, diagnosis, monitoring, and treatment. This is indeed a good move in decreasing the prevalence of mental health concerns and further favors the issue's importance. Studies that are being conducted in this regard yield no difference in results between in-person therapy and the use of innovative smartphone applications [[1], [2], [3], [4]]. But there are a lot of limitations to the results, including reliance on patient feedback for estimation of outcomes which are themselves kept unblinded during the study period; lack of scientific investigation of rationality and validity of the results these mobile applications calculate along with the usage of assessment scales for diagnosis monitoring and follow up. It further provokes confusion about the authenticity of the results of studies that have already been done in this regard and shows no difference.

So proper research should be conducted in this regard in the same way the drug trials are conducted before any new drug is approved to be utilized and made available to the general population. We further recommend welcoming an uprising of digital psychiatry but with caution; check and balance along with proper attentiveness to the fact that it doesn't become a slippery slope that leads to medical errors and loss of belief in the mental health care system worldwide that already is in the naive stages of gaining strength due to worldwide stigmatization of mental health concerns.

## References

[1] Krzystanek M., Borkowski M., Skałacka K., Krysta K. (2019 Feb). A telemedicine platform to improve clinical parameters in paranoid schizophrenia patients: results of a one-year randomized study. Schizophr. Res..

[2] Stolz T., Schulz A., Krieger T., Vincent A., Urech A., Moser C., Westermann S., Berger T. (2018 Jun). A mobile app for social anxiety disorder: a three-arm randomized controlled trial comparing mobile and PC-based guided self-help interventions. J. Consult. Clin. Psychol..

[3] Faurholt-Jepsen M., Lindbjerg Tønning M., Fros M., Martiny K., Tuxen N., Rosenberg N., Busk J., Winther O., Thaysen-Petersen D., Aamund K.A., Tolderlund L., Bardram J.E., Kessing L.V. (2021 May). Reducing the rate of psychiatric re-admissions in bipolar disorder using smartphones-The RADMIS trial. Acta Psychiatr. Scand..

[4] Tatham I., Clarke E., Grieve K.A., Kaushal P., Smeddinck J., Millar E.B., Sharma A.N. (2022 Mar 23). Process and outcome evaluations of smartphone apps for bipolar disorder: scoping review. J. Med. Internet Res..

